# Supplementary material for: Patterns of Intron Gain and Loss in Fungi
Source: PLoS Biol. 2004 Nov 30;2(12):e422. doi: 10.1371/journal.pbio.0020422 (PMC532390; doi:10.1371/journal.pbio.0020422)
Supplement: Table S1 — Also available at http://genes.mit.edu/NielsenEtAl/. (4.3 MB ZIP). [file pbio.0020422.st001.zip › NielsenEtAl/html/300.html]

AN4168.1.NCU03888.1.MG02506.1.FG09497.1


```
 CLUSTAL W (1.82) Multiple Sequence Alignments - Introns Inserted


Sequence 1: NCU03888.1	413 aa
Sequence 2: MG02506.1	388 aa
Sequence 3: FG09497.1	401 aa
Sequence 4: AN4168.1	458 aa
Alignment Length: 468 aa
Number Identitical Residues: 196 aa
Alignment Score (without introns) 9444


MG02506.1 	--MGINNPLPSSMAS1ECKKCGKILASFIDPRQAYGPD-----------K0GLAILTVIK
NCU03888.1	--MGINNPLPSSLSS1ECKKCGKILASFIEPKQAFGPDKVIPPSVLANAK0GLAILTVIK
FG09497.1 	--MGINNPLPSSMAS1ECKKCGKILESFINPRQSFGPDKVIPPSILANAK~GLAVITVLK
AN4168.1  	MPLGIHNPLPSSLSS1ECKKAGKILASFVDPRQAFGPDKVIPPEVLAGAK0GLAILTVLK
          	 .:**:******::* ****.**** **::*:*::***.  ...  :.:* ***::**:*

MG02506.1 	AGFVGSARFGSGLVIARLPDGSWSAPSAIATGGAGVGGQIGFELTDFVFILNDAAAVKTF
NCU03888.1	AGFLGSARFGSGLVVARLPDGSWSAPTAIATGGAGFGGQIGFELTDFVFILNDASAVKTF
FG09497.1 	AGFLGSGRFGSGLVVARLPDGGWSAPSAIATAGAGFGGQIGFELTDFVFILNDASAVRTF
AN4168.1  	AGFLGSGRFGSGIVVARLADGSWSAPSAIATAGAGFGGQIGFELTDFVFILNDAAAVRTF
          	***:**.*****:*:***.**.****:****.***.******************:**:**

MG02506.1 	SQSGSLTLGGNVSIAAGPVGRNAEAAGAASLRSVAGIFSYSKTKGLFAGVSLEGSAIVER
NCU03888.1	AQAGSLTLGGNVSVAAGPVGRNAEAAGAASLRSVAGIFSYSKTKGLFAGVSLEGSMIIER
FG09497.1 	AQAGSLTLGGNVSLAAGPVGRNAEAAGAASLKGVAGIFSYSKTKGLFAGVSLEGSAIIER
AN4168.1  	SQAGTLTLGGNVSIAAGPVGRNAEAAGAASTKGVAAVFSYSKTKGLFAGVSLEGSMLVER
          	:*:*:********:**************** :.**.:****************** ::**

MG02506.1 	KDANTKMYGRPVSAQELLTGGERAPPAAAPLINILTSRAFSGAGRSGSFNDGMYNDTPQY
NCU03888.1	KDANAKLYGRQVSAKELLSGAERPPPQAAPLLSILNSRIFAGMS-SGSFDDRMYNDQPVY
FG09497.1 	RDANEKMYGQRFTAQQLLTGSVRPPPQAAPLMNILNSRAFGGMR--GGNDDAMYNDAPVY
AN4168.1  	RDANERLYNSRVSARQLLSGTIPPPPAAEPLMRVLNSRAFYGVR---TNGDSMYNDIPVY
          	:*** ::*.  .:*::**:*   .** * **: :*.** * *       .* **** * *

MG02506.1 	DSHRDHVVWEGRRGSAYGETPSTRDAGAGAS---------EFGAPKRSATWQHDTYNNND
NCU03888.1	DNTHDSVVWGDRTGNAYGEG-QTRDSGYGASGGSGGQAHDEFGRPKRASTWQDDVYDQQP
FG09497.1 	DESHDDVVWNGRRGSAMGEGVQRNNTGLQHD---------SFGRPSRSSTWQDDVYDRQG
AN4168.1  	DDRHDDVVWEGRRGEAYGEGIRRDRTGYHGPS----DDYEYHDRPRRATTWADDIYDRPA
          	*. :* *** .* *.* **      :*    .    .  . .. * *::** .* *:.  

MG02506.1 	GYQSPRFGAAGISRSNTVNDARTTSAFGAPAAGGVAGSR--GPKPPVAPKPNFSNKQ---
NCU03888.1	RGFSNNRASVQYGR-NDTNGGGFGGGYGAAAAQQAAADKKVGPGRPAAPKPNYASKQ---
FG09497.1 	GYGGPTRSNTVASNGGAAND----YVYRDSPAGGFGAEKKAGPGRPAAPKPNFGAKQ---
AN4168.1  	GGLS-RSSTARFSSRNDTFDTYNRQRSNTYDDDYVYSDRK--PSRPTAPKPVFGQRTGQA
          	   .   . .  .  . . .                ..:.  *  *.**** :. : ..:

MG02506.1 	AMLQKNQAVALFTFEADQPGDLGFKKGEIITVVKKTDSDNDWC2YLSTVTVTRRLNG~D-
NCU03888.1	ALMKKNEAVALYTFNADQPGDLGFQKGEIITILKKTDSDNDWW2TGMIGTRHGTFPS~NY
FG09497.1 	AMLKKNEAVAVYNFDADQPGDLGFKKGDVITVLKRTESENDWW2TGQIGTRTGIFPS~NY
AN4168.1  	APLREDQAIALYTFDADQDGDLGFKKGEIITIIKRTEKKEDWW~TGRIGDRVGIFPA2FS
          	* :::::*:*::.*:*** *****:**::**::*:*:..:**            : .   

MG02506.1 	----------------------------------------------------
NCU03888.1	VKMIE-----------------------------------------------
FG09497.1 	VKMKE-----------------------------------------------
AN4168.1  	LIQACLGEINIKEVDKRHLQVILAVVEAGTRHLQGEEIWASAGLRPVESGKR
          	      .. . .. ..   .   :  .:.:   ....  :::.  . .:..
```
